# Pregnancy after Double Ovariotomy

**Published:** 1902-06-21

**Authors:** 


### PREGNANCY AFTER DOUBLE OVARIOTOMY.

At the last meeting of the Obstetrical Society, a case was reported by Mr. Alban Doran in which pregnancy had occurred after removal of both ovaries for cystic tumour. Mr. Doran said that, although it was not difficult to extirpate all ovarian tissue when the tumour was pedunculated, the case was different when the base of the cyst burrowed and lay close to the uterus. The same difficulty arose also with inflamed adherent appendages, and very often also with an ovary removed to check the growth of a uterine fibroid. The operator dared not cut the pedicle sufficiently short to remove all ovarian tissue, for he knew that if he did so the ligature would probably slip. Hence it was not very hard to understand why in some cases menstruation or even pregnancy occurred after removal of both ovaries. In his own case the ovarian ligament, with some healthy tissue, had probably been flattened against the uterus, and had thus escaped the ligature. This case, however, and others mentioned in the course of the discussion, raised the very interesting question how far the application of a ligature to both Fallopian tubes could be trusted to ensure sterility. Several cases were mentioned which went to show that the mere application of a silk ligature to the Fallopian tubes could not be depended upon to permanently obliterate their lumina, and thus prove an obstacle to pregnancy. Mr. Bland-Sutton said that when it was desirable to sterilise a patient, he always tied the tubes in two places and exsected a piece of the tube. Dr. Galabin, when the same object was desired, adopted the plan of cutting a piece out of the tube between two ligatures, then pulling out the stump on each side as far as possible, and cutting it off so that the open lumen was left at the bottom of an inverted cone of cellular tissue. None of the patients so treated had become pregnant again, and he was inclined to regard this as a reliable method.

